# Traumatic brain injury induces TDP-43 mislocalization and neurodegenerative effects in tissue distal to the primary injury site in a non-transgenic mouse

**DOI:** 10.1186/s40478-023-01625-7

**Published:** 2023-08-22

**Authors:** George R. Bjorklund, Jennifer Wong, David Brafman, Robert Bowser, Sarah E. Stabenfeldt

**Affiliations:** 1 School of Biological and Health Systems Engineering, Arizona State University, Tempe, AZ USA; 2 Department of Translational Neuroscience, Barrow Neurological Institute, Phoenix, AZ USA

**Keywords:** Traumatic brain injury, TBI, TDP-43, Neurodegeneration, ALS, Amyotrophic lateral sclerosis, Alzheimer’s disease, AD, Frontotemporal degeneration, FTD

## Abstract

Traumatic brain injury (TBI) initiates tissue and cellular damage to the brain that is immediately followed by secondary injury sequalae with delayed and continual damage. This secondary damage includes pathological processes that may contribute to chronic neurodegeneration and permanent functional and cognitive deficits. TBI is also associated with an increased risk of developing neurodegenerative diseases such as Alzheimer’s disease (AD), frontotemporal dementia (FTD), and amyotrophic lateral sclerosis (ALS) as indicated by shared pathological features. For example, abnormalities in the TAR DNA-binding Protein 43 (TDP-43) that includes cytoplasmic mislocalization, cytosolic aggregation, and an increase in phosphorylation and ubiquitination are seen in up to 50% of FTD cases, up to 70% of AD cases, and is considered a hallmark pathology of ALS occurring in > 97% of cases. Yet the prevalence of TDP-43 pathology post-TBI has yet to be fully characterized. Here, we employed a non-transgenic murine controlled cortical injury model of TBI and observed injury-induced hallmark TDP-43 pathologies in brain and spinal cord tissue distal to the primary injury site and did not include the focally damaged tissue within the primary cortical injury site. Analysis revealed a temporal-dependent and significant increase in neuronal TDP-43 mislocalization in the cortical forebrain rostral to and distant from the primary injury site up to 180 days post injury (DPI). TDP-43 mislocalization was also detected in neurons located in the ventral horns of the cervical spinal cord following a TBI. Moreover, a cortical layer-dependent affect was identified, increasing from superficial to deeper cortical layers over time from 7 DPI up to 180 DPI. Lastly, RNAseq analysis confirmed an injury-induced misregulation of several key biological processes implicated in neurons that increased over time. Collectively, this study demonstrates a connection between a single moderate TBI event and chronic neurodegenerative processes that are not limited to the primary injury site and broadly distributed throughout the cortex and corticospinal tract.

**Supplementary Information:**

The online version contains supplementary material available at 10.1186/s40478-023-01625-7.

## Introduction

The vast majority of age-related neurodegenerative diseases have no familial or genetic basis and are considered sporadic in nature due to complex genetic and environmental factors. For example, sporadic amyotrophic lateral sclerosis (ALS) occurs in approximately 90–95% of cases, frontotemporal dementia (FTD) is sporadic in approximately 70% of cases, and Alzheimer’s disease (AD), the most common neurodegenerative disorder, is defined as sporadic in up to 90% of cases [1–3]. Examples of non-genetic environmental factors that lead to a higher risk of developing neurodegenerative diseases include intense physical activity, traumatic brain injury (TBI), repeated TBI, chemical exposure, and certain health conditions [4, 5]. Specifically, TBI is increasingly associated with the future development of neurodegenerative diseases such as AD, FTD, and ALS [6].

TBI alone is a significant health problem with over 69 million annual cases reported globally [7]. The initial mechanical insult from a TBI causes an immediate primary injury including necrosis, edema, and blood–brain barrier disruption thereby initiating a multitude of secondary pathological molecular sequalae [8, 9]. This phase of injury response can include oxidative stress, excitotoxicity, metabolic, and mitochondrial dysfunction, alterations in protein synthesis, folding, and degradation, disruptions in cellular signaling, DNA damage, necrosis, and apoptosis [8]. It is well established that these secondary injury processes may contribute to sustained and chronic functional and cognitive deficits, observed in the clinical and pre-clinical models [10–13, 6, 14, 15]. Yet, A critical gap in knowledge in the neurotrauma community is elucidating the underlying mechanisms by which TBI may induce and/or contribute neurodegenerative-related pathologies, neuronal dysfunction, and cognitive decline.

Among pathologies found within individual neurodegenerative diseases, proteinopathies of the TAR-DNA binding protein 43 (TDP-43) are a common factor. TDP-43 is an RNA binding protein involved in a variety of critical cellular processes including RNA biogenesis and processing and the regulation of mRNA alternative splicing. Proteinopathies of TDP-43 include mislocalization from the nucleus, insoluble cytosolic aggregation, hyperphosphorylation, and increased ubiquitination [16, 17] observed throughout the central nervous system (CNS). Specifically for ALS, TDP-43 proteinopathies are a hallmark pathology and found in up to 97% of cases [18, 14]. The incidence for TDP-43 pathology in FTD and AD are ~ 50% and up to 70% of severe cases, respectively [18, 19, 14, 20].

Clinical and post-mortem studies in humans have increasingly demonstrated links between incidences of TBI and TDP-43 pathologies [21–25]. Much of the current post-mortem analyses center on chronic traumatic encephalopathy (CTE), a progressive neurodegeneration disease caused by repetitive mild traumatic brain injuries, rather than individual TBI events followed by neurodegenerative diseases. However, within these CTE cases, a strong link between CTE and TDP-43 proteinopathies has been established. For example, in 2010 McKee et al., found that 10 out 12 athletes that were diagnosed with CTE, also showed widespread TDP-43 proteinopathies [26], thereby indicating a potential link between TBI and TDP-43-related neurodegeneration.

Outside of post-mortem CTE cases, human population-based studies have also been performed to determine a link between a prior TBI and subsequent neurodegeneration. In a 2018 meta-analysis of studies, Huang et. al., concluded that TBI is a potential risk factor for certain neurodegenerative diseases as TBI patients frequently exhibited neurodegenerative processes such as behavioral, cognitive, and/or motor-related symptoms [27]. A major limitation however is this study included a variety of severities in the initial TBIs. Although the data only included patients that sought treatment for TBIs and neurodegenerative disease, it would still be difficult to discern a relationship between TBI severity or type and neurodegenerative disease.

In preclinical studies using studies using different models of TBI, injury severities, and varying animal species, both transgenic and wild-type, have previously described TDP-43 mislocalization and related pathologies [28–33]. However, these earlier studies focused on TDP alterations at or within proximity to the primary site of injury, and acutely following TBI. Further characterization of the areas distal to the primary injury site, as well as areas along the connecting corticospinal tract long-term post-TBI would serve to help fully assess neurodegenerative effects following an experimental TBI.

To address the gaps in understanding the chronic neurodegenerative TDP-43 pathology following a TBI, here, we employed a unilateral controlled cortical impact (CCI) model in non-transgenic C57BL/6J mice with no predisposition to neurodegenerative diseases. Age-matched naïve control animals (no sham surgeries) were used for all comparisons as sham surgeries in themselves may result in measurable cortical injury and would not allow for baseline non-injured control comparisons [34]. The primary cortical injury was located over the primary motor cortex and somatosensory cortex thus affecting the intracortical as well as subcortical pathways. Immunohistological tissue analysis (IHC) and RNAseq was performed at 7, 14, 28, 120, and 180 days post-injury (DPI). We focused our analysis on functionally connected cortical regions distal from the injury primary site (i.e., regions without a visible cortical lesion cavity) and the functionally connected cervical spinal cord; these sites were verified using the Allen Reference Atlas, specifically the C57/BL6 mice and the MouseLight project at Janelia [35, 36]. Significant TBI-induced neuronal TDP-43 mislocalization located in the forebrain and cervical spinal cord distal to the primary injury site was observed over the course of the study (out to 180 DPI). Moreover, TDP-43 mislocalization significantly increased with time post-injury up to 180 DPI. This temporal increase in cortical TDP-43 mislocalization post-injury was layer-specific progressing from superficial cortical layers to deeper cortical layers over the analysis time course. Finally, RNA-seq analysis of cortical and cervical spinal cord tissue showed a similar pattern of significantly increasing transcriptional misregulation over the course of the study. Furthermore, significant transcriptional misregulation was evident in numerous biological processes that are associated with neurodegenerative diseases [37, 38]. Our findings demonstrate that a single unilateral TBI induced significantly increasing and chronic neuronal TDP-43 mislocalization as well as significant transcriptional misregulation in CNS tissues distal to the primary injury site.

## Materials and methods

### Animals

All animal experiments were performed in accordance with established procedures approved by the Institutional Animal Care and Use Committee of Arizona State University and NIH guidelines for the use and care of laboratory animals. Mice used in this work were non-transgenic C57BL/6J males from The Jackson Laboratory (RRID:IMSR_JAX:000664) and housed in standard conditions. Male mice were used exclusively in this study to reduce any confounding effects due to sex differences in both the TBI and detectable neurodegenerative effects. Future studies to account for variables due to differences in sex are planned to supplement and enhance the findings of this study. The mice were kept on a 12-h light/dark cycle with food and water provided ad libitum. Age-matched naïve controls (no sham surgeries) were used for each timepoint analyzed with the exception of the early timepoints of 7, 14, and 28 DPI that used a common age-matched control.

### Unilateral controlled cortical impact (CCI)

Unilateral CCIs were performed on mice in the right frontoparietal cortex as previously described and by the following detailed procedure [39–41]. Mice were anesthetized with 1.5–3.0% isoflurane gas and placed in a mouse stereotaxic frame. The surgery site was prepared by shaving followed by a betadine scrub and isopropyl rinse. A homeothermic warming system was used to maintain mouse body temperature during the surgical procedure. An incision was made along the midline from behind the eyes to the back of the skull and a 3 mm craniotomy was performed approximately 1.5 mm posterior to Bregma and 1.5 mm lateral from midline with a fresh biopsy punch. The Leica Impact One CCI device with a 2 mm probe was positioned to the center of the craniotomy and perpendicular to the exposed brain. The CCI was performed at 6.0 m/s for a duration of 100 ms and a cortical depth of 1.0 mm, modeling a moderate TBI. Following the CCI, any bleeding was stopped and the surgery site cleaned before replacement of the craniotomy bone flap and securement with sterile dental adhesive. Skin closure was performed with 4–0 absorbable sutures and a triple antibiotic ointment was applied to the suture site. Animals were then given a subcutaneous injection of analgesia (buprenorphine; 0.05 mg/kg) and sterile saline (0.5mls). Finally, the animals were placed in a fresh cage on a heating pad and monitored for 1–2 h before being placed in the vivarium for post-operative monitoring and care.

### Tissue preparation

Animals were given a lethal dose of sodium pentobarbital (200 mg/kg Euthasol, i.p.) then transcardial perfusions were performed on mice by first clearing with ice-cold PBS then fixing with a 4% paraformaldehyde/PBS (PFA) solution. Dissected brains were post-fixed in PFA the solution overnight at 4 °C then serially incubated in 15% and 30% sucrose/PBS solutions prior to embedding in Tissue-Tek O.C.T. (Tissue-Tek 4583) and freezing at −80 °C. Cryostat sectioning was performed with sections being collected on Fisherbrand Superfrost/Plus slides (Fisher Scientific 12-550-15) and air-dried prior to staining. Alternatively, 30–50 µm cryostat sections were serially collected in 6-well culture plates with cold PBS for free-floating staining.

### Immunohistochemistry

Tissue sections were permeabilized with PBS/0.3% Tween-20 (PBST) for ~ 30 min then blocked with 5% normal donkey or normal horse serum in 0.01% PBST at room temperature for ~ 1 h. Primary antibodies were diluted in 0.01% PBST and 5% serum then incubated overnight at 4 °C with gentle shaking. Primary antibodies used for this study were; rabbit anti-TARDBP (Proteintech 10,782–2-AP), mouse anti-NEUN (Millipore Sigma MAB377), and DAPI (4′,6-diamidino-2-phenylindole)(Sigma Aldrich 10236276001). After rinsing in PBST 3X, secondary antibodies diluted in 0.01% PBST and 5% serum were added and incubated at room temperature for ~ 1 h with gentle shaking. Secondary antibodies included Alexa Fluor 555 anti-rabbit (Abcam ab150106) and Alexa Fluor 647 anti-mouse (Jackson ImmunoResearch 715-605-151). DAPI was used for nucleus staining. Imaging was performed using a Zeiss LSM 800 laser scanning confocal microscope (LSCM) with Airyscan.

### Image analysis and quantitation

#### Cortical neuronal counts and TDP-43 mislocalization quantitation

Quantitation of cortical neuronal bodies exhibiting TDP-43 mislocalization was performed by selecting a radial column of the cortex at approximately Bregma 1.1 encompassing all cortical layers within portions of primary and secondary motor areas of the frontal cortex separate from the injury area (ROI) [42]. Both ipsilateral and contralateral (to unilateral injury site) cortical layers (2/3, 5, and 6) were quantified separately and was performed by an investigator blinded to the group (injured or naïve). The ROI containing only NEUN-positive labeled neurons was selected in Adobe Photoshop and transferred to Imagej [43]. In Imagej, pixel intensities were adjusted with either the Mean or MinError auto threshold component, smoothed, holes filled, and watershed treated prior to auto cell counting and outline mask creation. The outline mask was then transferred to the original image in Photoshop where the identified and counted NEUN-positive neurons were examined for TDP-43 mislocalization and counted. Counts were then calculated to provide the total number of NEUN-positive neurons and the percentage of NEUN-positive neurons that displayed evidence of TDP-43 mislocalization.

Representative images were cropped and adjusted for brightness and contrast in Photoshop for presentation. All images used for comparison analysis were collected using the same microscope settings and all adjustments made were done equally between images. Unstained tissue as well as secondary only controls were utilized to verify appropriate laser and fluorophore illumination settings.

#### Cervical spinal cord neuronal counts and TDP-43 mislocalization quantitation

Neuronal bodies, primarily interneurons and Alpha motor neurons, were quantified in the ventral horns of the cervical spinal cord at approximately C7-C8. The ROI was outlined for each hemisphere from the midline of the spinal cord and the bottom of the dorsal corticospinal tract. NEUN-positive neurons were quantified and examined for those that displayed indications of TDP-43 mislocalization. Counts were then calculated to provide the total number of NEUN-positive neurons and the percentage of NEUN-positive neurons that displayed evidence of TDP-43 mislocalization.

### Statistical analysis

Statistical analysis was performed in Graphpad Prism Ver. 9.3.1 (GraphPad Software, San Diego, CA). Direct comparisons between cortical or spinal hemispheres and specific time point injured vs control comparisons were performed by two-tailed t-test using Welch’s correction for unpaired and differing standard deviations. Analysis of TDP-43 mislocalization over time was performed by two-way ANOVA using Bonferroni's multiple comparisons test. Results were considered significant with a *p*-value ≤ 0.05 and were reported where appropriate. Mean and standard error of the mean shown. All statistical measurements included in Additional File 1: Table S1 through 11.

### RNA isolation and purification

Tissue used for RNA isolation consisted of cortical tissue rostral to and separate from the initial injury site and cervical spinal cord tissue approximately C2 to T2. Cervical spinal cord tissue and cortical tissue from the same set of mice were used in the cortical RNAseq analysis. RNA isolation and purification was performed using Qiagen RNeasy Lipid Tissue Mini Kits with approximately 80–90 mg tissue for each sample per the manufacturers’ directions. Briefly, Qiazol lysis reagent was added to neural tissue in a 2 ml Dounce homogenizer to extract total RNA. Chloroform was added to the homogenized tissue and mixed thoroughly then centrifuged to separate the mixture to three separate phases. The aqueous phase containing the extracted RNA was removed to a new tube and the remaining was saved for purification of cellular proteins. The RNA extract was then purified in accordance with all Qiagen procedures except that centrifuge speeds and time were increased and a final wash of the membrane was performed using 80% ethanol and a final drying spin at max speed for 5 min.

### mRNA sequencing and analysis

Functional profiling of GO biological processes were determined using PANTHER tools (ver. 17.00) [44]. Input for analysis used differentially expressed genes that were filtered to use only those with a padj value < 0.05 and log2 fold change > 0.585 (± 1.5 fold change) unless specified. Predicted genes and RIKEN cDNA were excluded from all analysis. FASTQ files from RNA sequencing were aligned to the mouse genome, Mus musculus GRCm38/mm10 using HISAT2 (version 2.0.5). Differential analysis was performed using DESeq2 (version 1.20.0) and edgeR (version 3.22.5), with log2(Fold Change) >  = 1 and padj <  = 0.05. Enrichment analysis was performed using clusterProfiler (version 3.8.1) with a padj < 0.05.

## Results

### TBI induces nucleocytoplasmic mislocalization of TDP-43 in the cortex

**Fig. 1:**
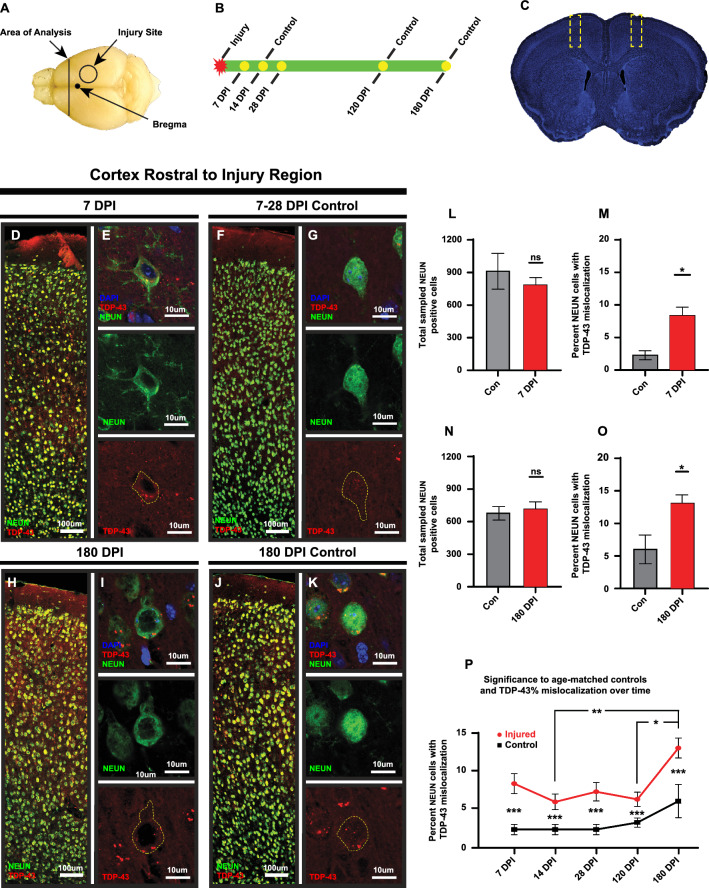
TDP-43 is mislocalized in cortical neurons distal from the injury site following a TBI. **A** Cortical analysis area shown in relation to the unilateral injury site and Bregma. **B** Sample tissue collection timeline post injury including age-matched naïve controls. **C** Outlines of cortical areas used for cell count analysis. **D**–**G** and **H**–**K** Representative images of total cortical areas analyzed for 7 and 180 DPI injured and naïve controls (respectively) including hi-res sample images of neurons displaying TDP-43 mislocalization (injured samples) and those that do not (control samples). **L**–**O** Total number of sampled NEUN-positive neurons for 7 and 180 DPI timepoints showed no significant differences between injured and naïve controls (*p* = 0.4796 and *p* = 0.6938 respectively) while the percent of NEUN-positive neurons that display TDP-43 mislocalization differed significantly (*p* = 0.0002 across samples). **P** Percentage of NEUN-positive neurons that display TDP-43 mislocalization between injured and naïve control animals over all analyzed timepoints (7–180 DPI ns, 14–180 DPI *p* = 0.0088, 28–180 DPI ns, 120–180 DPI *p* = 0.0210). Significance marked below error bars is between age-matched injured and naïve control animals and was found to be *p* = 0.0002 for all comparisons. Non-significant comparisons between timepoints not shown. (Mean with standard error from the mean used for error bars) (Significance: *p < 0.05, **p < 0.01, ***p < 0.001, ****p < 0.0001)

To examine the effect of TBI in the context of a non-transgenic animal model, we employed a controlled cortical impact (CCI) model in 9–10 week old male wild-type C57BL/6J mice (Fig. 1A). To assess the extent to which CCI modulates observed pathologies over time, animals were examined at 7, 14, 28 DPI (subacute) and 120 and 180 DPI (chronic) timepoints and compared to naïve age-matched control animals (Fig. 1B). Sampled areas are within the frontal cortex in both primary and secondary motor areas, rostral and distal to the primary injury site and included all cortical layers. Areas of analysis were distal from the injury primary site and without a visible cortical lesion cavity (Fig. 1A, C).

We first analyzed NEUN-positive neuron (RBFOX3, RNA Binding Fox-1 Homolog 3) counts in both ipsilateral and contralateral hemispheres and found no significant differences in total NEUN-positive neurons between hemispheres (Fig. 1L, N) (Additional File 1: Table S1). We next examined cortical tissue samples for intracellular TDP-43 mislocalization. TDP-43 is an RNA-binding protein that is predominantly found in the nucleus [45], as demonstrated by the representative immunostaining in the naïve animals (Fig. 1G, K). By comparison, cytoplasmic accumulations and mislocalization of TDP-43 were observed in cortical neurons from injured animals from 7 to 180 DPI (Fig. 1D, E, H, I). Cytoplasmic TDP-43 mislocalization in NEUN-positive neurons was observed in 2.44% ± 0.69% (SEM) of cortical neurons from naïve control animals and significantly increased to 8.55% ± 1.33% (SEM) in injured animals at 7 DPI (t(27) = 5.763, *p* = 0.0002) (Fig. 1M; Additional File 1: Table S2). Similarly, at 180 DPI injured animals had a significantly higher percentage of NEUN-positive cortical neurons with TDP-43 mislocalization, 13.27% ± 1.32% (SEM), than age-matched naïve controls, 6.22% ± 2.21% (SEM) (t(27) = 5.763, *p* = 0.0002) (Fig. 1O; Additional File 1: Table S2). Furthermore, these significant increases between injured and naïve control mice were detected at all timepoints in our analysis (Fig. 1P; Additional File 1: Table S2). Importantly, this analysis revealed that there is no statistically significant differences between total sampled NEUN-positive neuron counts of the injured and age-matched controls at all time points analyzed with all *p* values > 0.9999 (t(1.23) = 27.000) (Fig. 1L, N; Additional File 1: Table S3). Furthermore, analysis of the percent of NEUN positive cells displaying TDP-43 nucleocytoplasmic mislocalization between ipsilateral and contralateral cortices revealed no significant differences (Additional File 1: Table S4).

Next, we probed whether the persistence of NEUN-positive neurons with TDP-43 cytoplasmic accumulation increased with time post-injury. Two-way ANOVA revealed significant differences between the 14 DPI and 180 DPI timepoints and the 120 DPI and 180 DPI timepoints in the injured animals (t(5.763) = 27, *p* = 0.0002) (Fig. 1P; Additional File 1: Table S5). However, no significant differences were found between any other time points in injured animals (i.e., 7 vs. 14 DPI, 7 vs. 28 DPI, etc.). In contrast, the naïve age-matched control animals exhibited no temporal increase in NEUN-positive neurons with TDP-43 cytoplasmic accumulation (Fig. 1P).

These results show that that a single TBI induced acute dysregulation of TDP-43 leading to mislocalization that persists at a sustained level out to 120 DPI. This TDP-43 cytoplasmic accumulation then significantly increased by 180 DPI compared to this initial acute/subacute level.

### TDP-43 mislocalization in response to TBI is cortical layer specific

**Fig. 2:**
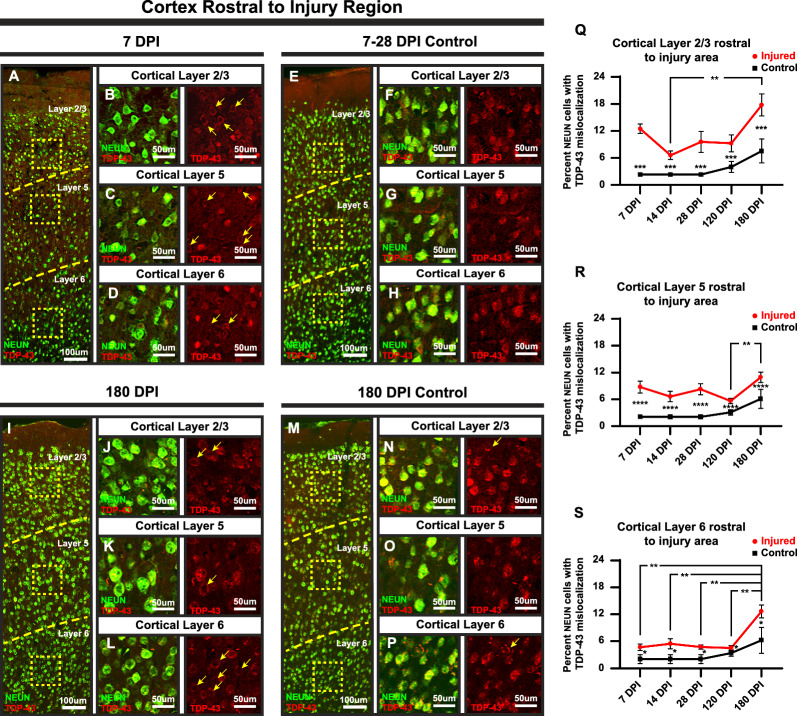
TDP-43 mislocalization displays changes in a layer specific manner over time. **A**–**H** and **I**–**P** Representative images of quantified cortical columns with representative layer divisions and insets of assessed layers, 2/3, 5, and 6, for 7 and 180 Dpi and age-matched controls. **Q** Increases in TDP-43 mislocalization in layer 2/3 of the injured mice shows no significant difference between 7, 28, and 120 DPI and 180 DPI timepoints but does show a significant increase from 14 to 180 DPI (14–180 DPI *p* = 0.0055). Significance marked below error bars is between age-matched injured and naïve control animals (*p* = 0.0002 across samples). **R** In layer 5, the 120 DPI timepoint displayed a significant change when compared to the 180 timepoint DPI (*p* = 0.0393). Significance marked below error bars is between age-matched injured and naïve control animals (*p* =  < 0.0001 across samples). **S** All timepoint comparisons in layer 6 showed significance when compared to the 180 DPI timepoint (7–180 DPI *p* = 0.0029, 14–180 DPI *p* = 0.0076, 28–180 DPI *p* = 0.0031, 120–180 DPI *p* = 0.0075). Comparisons between injured and naïve controls showed a significant difference at for all timepoints (*p* = 0.0234 across all timepoints). Non-significant comparisons between timepoints not shown. (Mean with standard error from the mean used for error bars) (Significance: *p < 0.05, **p < 0.01, ***p < 0.001, ****p < 0.0001)

Previous studies have shown that TBI may differentially affect the various layers of the cortex resulting in cognitive and functional deficits due to functional changes in neurons such as dendritic degeneration, synapse reduction, or inhibitory/excitatory fluctuations [46–48]. To that end, NEUN-positive neurons were analyzed for TDP-43 mislocalization within cortical layers 2/3, 5, and 6 in areas of the primary motor cortex. As previously described, cortical areas for this analysis were rostral and distal to the injury site and did not include tissue from the injury area. Two-way ANOVA Comparisons of injured and naïve age-matched controls revealed significant differences at each analyzed timepoint in each of the analyzed cortical layers as can be seen in the representative images (Fig. 2A–H 7DPI, and I–P 180 DPI), the accompanying graphs (Fig. 2Q–S), and Additional File 1: Tables S1, S6, S7 and S8.  Visual comparisons at the 180 DPI timepoint revealed a more prominent presentation of cytoplasmic TDP-43 accumulations in the injured mice when compared to the age-matched naïve control mice (Fig. 2I–L and M–P). This TDP-43 cytoplasmic accumulation also appears more prominent when compared to the 7 DPI injured and age-matched naïve control mice (Fig. 2A–D and E–H).

Assessment of the temporal profile of NEUN-positive neurons with TDP-43 mislocalization across the analyzed cortical layers revealed differing patterns from the 7 DPI timepoint up to the 180 DPI timepoint in the injured mice (Fig. 2Q–S; Additional File 1: Tables S6, S7, S8). While there appeared to be an appreciable difference between all analyzed timepoints and the 180 DPI timepoint in layers 2/3 and layer 5, only the 14 DPI timepoint in layers 2/3 and the 120 DPI timepoint in layer 5 reached statistical significance (t(27) = 4.479, *p* = 0.0055 14 to 180 DPI layer 2/3, t(31) = 3.683, *p* = 0.0393 120 to 180 DPI layer 5) (Fig. 2Q–R; Additional File 1: Tables S6 and S7). In comparison, all analyzed timepoints in layer 6 injured mice showed statistical significance when compared when compared to naïve age-matched control mice (t(31) = 3.872, *p* = 0.0234) (Fig. 2S; Additional File 1: Table S8).

Collectively, these results show layer-specific differences in injury-induced TDP-43 mislocalization. Of particular note when comparing the analyzed layers is the pattern of TDP-43 mislocalization at the earlier timepoints (i.e., 7–120 DPI) (Fig. 2Q–S). While these earlier timepoints show higher to moderately higher increases in layers 2/3 and 5, layer 6 shows little, yet significant, increases until the TDP-43 pathology strikingly emerges at 180 DPI.

### TDP-43 mislocalization is observed in the cervical spinal cord following a unilateral cortical TBI

**Fig. 3:**
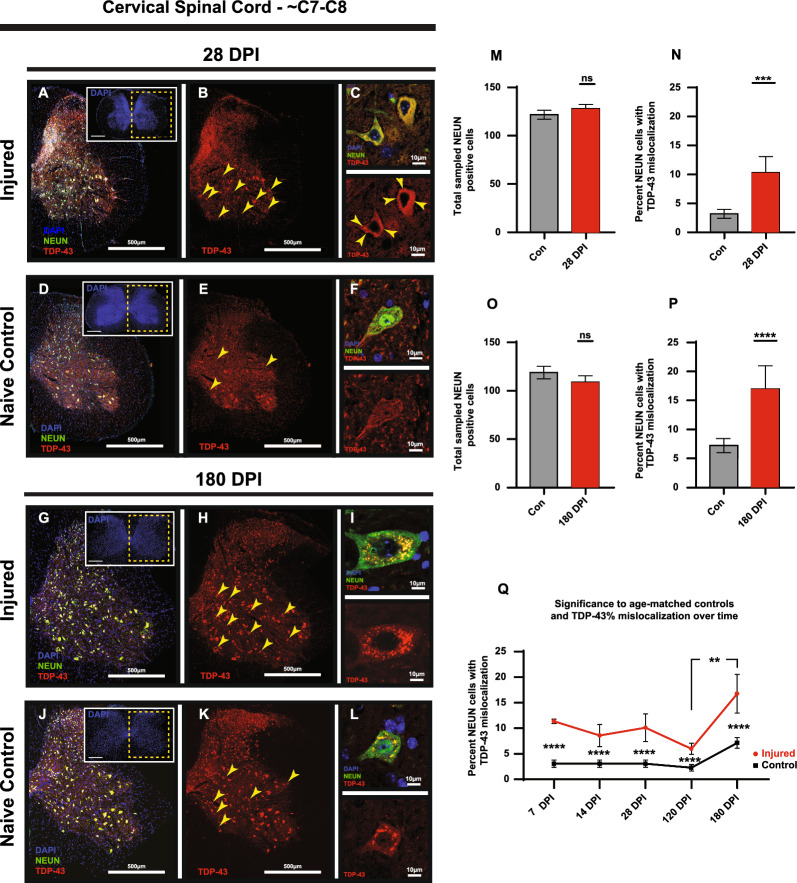
TDP-43 is mislocalized in NEUN-positive neurons of the cervical spinal cord following a unilateral TBI. **A**–**F** and **G**–**L** Representative images of 28 DPI and 180 DPI injured and naïve age-matched controls illustrating TDP-43 nuclear mislocation in the cervical spinal cord ventral horns. Note the discernable differences in cytosolic accumulations of TDP-43 between the two samples shown in the hi-res images at 180 DPI. **M**, **N** Total sampled NEUN-positive neurons showed no significant differences between injured and naïve at 28 DPI. NEUN-positive neurons that displayed TDP-43 mislocalization however displayed a significant increase between injured and naïve samples (*p* = 0.0001). **O**, **P** 180 DPI samples also showed no significant differences in overall NEUN-positive neuron counts with a significant difference of NEUN-positive neurons displaying TDP-43 mislocalization when compared to naïve age-matched controls with (*p* =  < 0.0001). **Q** Percentage of NEUN-positive neurons that display TDP-43 mislocalization between injured and naïve age-matched control animals compared to the 180 DPI timepoint showed no significant differences until the 120 DPI to 180 DPI timepoint (*p* = 0.0045). Significance marked below error bars is between age-matched injured and naïve control animals (*p* =  < 0.0001 across all timepoints). Non-significant comparisons between timepoints not shown. (Mean with standard error from the mean used for error bars) (Significance: *p < 0.05, **p < 0.01, ***p < 0.001, ****p < 0.0001)

To determine whether injury-induced TDP-43 mislocalization was also present in neurons further distally within the corticospinal tract, we analyzed NEUN-positive neuronal bodies in the ventral horns of the cervical spinal cord at C7-C8. Similar to our studies in the cortex, no significant differences were found in NEUN-positive neuronal counts between ipsilateral and contralateral hemispheres (Fig. 3M, O; Additional File 1: Table S9). Also similar to the cortical analysis, the percentage of NEUN positive cells that display TDP-43 nucleocytoplasmic mislocalization showed no significant differences between spinal cord hemispheres (Additional File 1: Table S10). Immunohistochemical analysis revealed that TDP-43 mislocalization in NEUN-positive neurons was present in the cervical spinal cord of injured mice at sub-acute (i.e., 7 through 28 DPI (28 DPI shown)) (Fig. 3A–F and M–N) and chronic (i.e., 180 DPI; Fig. 3G–L and O–P) time points post-injury when compared to naïve age-matched control mice (t(33) = 6.151, *p* =  < 0.0001) (Fig. 3Q; Additional File 1: Table S11).

Over time, only the 120 DPI timepoint to the 180 DPI timepoint in injured mice reached significance (t(33) = 4.419, *p* = 0.0045) (Fig. 3Q; Additional File 1: Table S11). Importantly, the naïve age-matched control animals demonstrated no temporal changes in NEUN-positive neuron TDP-43 mislocalization.

These results indicate that a single TBI of functionally connected motor regions within the cortex induce significant temporal changes in TDP-43 mislocalization in NEUN-positive cells of the ventral horns in the cervical spinal cord.

### Injury induces time-dependent transcriptional changes in the frontal cortex

**Fig. 4:**
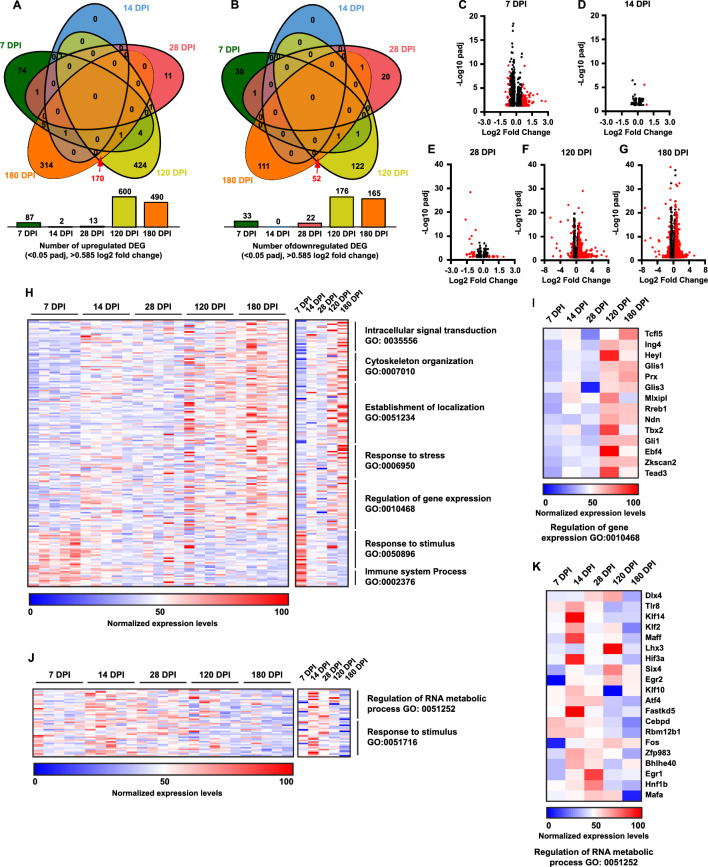
RNAseq analysis shows increasing misregulation of key biological processes in the cortex. **A**–**B** Venn diagrams illustrating the lack of DEG overlap between early and late timepoints for both upregulated and downregulated DEGs. **C**–**G** Volcano plots showing increase in DEGs that exceed a log2 fold change of 0.585 (1.5 fold change) with a padj value < .05 over the course of analysis. (Note missing points at 120 DPI: Wdfy1 log2 fold change = 1.27, padj −log 10 = 52.83, 180 DPI: Tmem181b and Gabra2 fold change = 1.58 and 1.02, padj −log 10 = 66.44 and 52.95 respectively). **H** Heat map with representative genes from listed significant GO biological processes. Data used for analysis is from the 180 DPI upregulated list except Response to stimulus and Immune system process which were taken from 7 DPI upregulated list. All samples shown for each time point. **I** Heat map of averages of all analyzed samples from each timepoint with attached gene list. **J** Heat map with representative genes from listed significant GO biological processes. Data used for analysis is from the 180 DPI downregulated list. All samples shown for each time point. **K** Heat map of averages for analyzed samples from each timepoint with attached gene list. Only DEGs with log2 fold change > 0.585 (1.5 fold change) and padj value < .05 used for this analysis. Cortical forebrain tissue analyzed included both ipsilateral and contralateral (to the unilateral TBI) tissue samples combined

To examine the impact of injury on the transcriptional profiles of the frontal cortex, we performed bulk RNA-sequencing (RNA-seq) on tissue collected from 7, 14, 28, 120 and 180 DPI as well as age-matched naïve animals. Differential gene expression (DEG) analysis was then performed to identify genes differentially up- or down-regulated in injured tissue compared to naïve tissue over time post-TBI. Broadly, this analysis revealed a higher number of differentially expressed genes at acute (i.e., 7 DPI; Fig. 4C) and chronic time points (i.e., 120, 180 DPI; Fig. 4F–G) post-injury when compared to sub-acute time points post-injury (i.e., 14, 28 DPI; Fig. 4D, E). Interestingly, only 1.29% of unique upregulated (Fig. 4A) and 0.88% of unique downregulated genes (Fig. 4B) were found to overlap between the acute/subacute and the chronic timepoints post-injury. This finding suggests that injury induces distinct transcriptional responses acutely post-TBI that then impacts the transcriptional profile chronically after TBI.

Gene ontology (GO) analysis then identified the biological processes that were modulated by injury at acute and chronic time points. Biological processes represented as significantly upregulated at the acute timepoint of 7 DPI included Response to Stimulus (GO:0050896) and Immune System Response (GO:0002376) (Fig. 4H). Each of these processes encapsulate several child GO processes and are defined as processes that that result in changes to the state or activity of cells (response to stimulus) and processes that are involved in immune system and immune system responses to potential threats (immune system responses). In comparison, the chronic timepoints of 120 and 180 DPI revealed significant upregulation of GO processes that involve biological processes such as Intracellular Signal Transduction (GO:0035556), Cytoskeleton Organization (GO:0007010), Establishment of localization (GO:0051234), Regulation of Gene Expression (GO:0010468), and Response to Stress (GO:0006950) (Fig. 4H). Highly represented downregulated biological processes at the 180 DPI chronic timepoint included Regulation of RNA Metabolic process (GO:0051252) and Response to Stimulus (GO:0051716) which is a child process of GO:0050896 that was upregulated at the 7 DPI acute timepoint.

This analysis demonstrates the early cellular responses to the initial injury were dominated by significant upregulation of immune and cellular response processes. However, over the course of six months in this study, these biological processes shifted acutely from response to chronic and significant misregulation of key cellular biological processes indicative of a progressive pathological process. Furthermore, these processes have been implicated as central players in neurodegenerative diseases [37, 38] further indicating a progressive neurodegenerative state over time following a TBI.

### RNAseq analysis reveals increased changes over time in the cervical spinal cord.

**Fig. 5:**
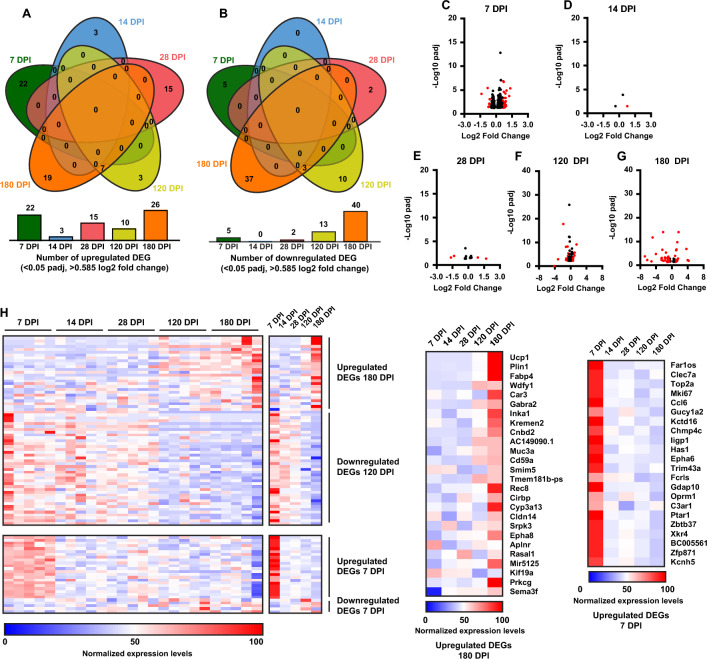
RNAseq analysis shows increasing misregulation of key biological processes in the cervical spinal cord. **A**–**B** Venn diagrams show the same lack of DEG overlap between the early and late timepoints in the analyzed spinal cord tissue as found in the cortex. **C**–**G** Volcano plots show a milder but still increasing amount of significantly misregulated genes that meet a log2 fold change > 0.585 (1.5 fold change) (Note missing points at 120 DPI: Wdfy1, Tmem181b-ps, Gabra2, and Kif5a, log2 fold change = 1.74, 1.41, 0.99, 1.15, and −0.34, padj −log 10 = 125.83, 89.76, 41.97, 40.37, and 25.87 respectively, 180 DPI: mt-Nd3 fold change = −3.96, padj −log 10 = 58.20). **H** Heat maps displaying changes over time of all up and downregulated DEGs for 180 and 7 DPI. **I**–**J** Heat maps of averaged samples from upregulated 180 and 7 DPI gene sets. Only DEGs with log2 fold change > 0.585 (1.5 fold change) and padj value < .05 used for this analysis. Spinal cord tissue included both ipsilateral and contralateral (to the unilateral TBI) tissue

To examine the impact of injury on the transcriptional profiles of the functionally connected cervical spinal cord (approximately C2-T7), we performed RNA-sequencing (RNA-seq) on tissue collected from 7, 14, 28, 120 and 180 DPI as well as age-matched naïve animals. Differential gene expression (DEG) analysis was then performed to identify genes differentially up- or down-regulated in tissue from injured animals compared to tissue from naïve animals over time post-TBI. Spinal cord analysis included only those genes that were differentially expressed with a log2 fold change > 0.585 (1.5 fold change) and a padj value < 0.05. Similar to the cortical analysis, VENN diagrams of up- and downregulated DEGs illustrate no relationship in DEGs between acute/subacute timepoints of 7, 14, and 28 DPI and chronic timepoints at 120 and 180 DPI (Fig. 5A, B). Additionally, much like the cortex, an increase in the number of significant DEGs is seen over the time post-injury as illustrated in both the VENN diagrams and volcano plots (Fig. 5C–G).

Due to the overall smaller compliments of differentially expressed genes, GO analysis revealed smaller groupings of significantly represented biological processes. Similar to the cortex analysis however, much of the same GO processes were represented over the course of analysis. These included biological processes that involved Signal Transduction (GO:00071650), Response to Stimulus (GO:0050896), Response to Stress (GO:0006950), Cell Communication (GO:0007154), and Protein Folding (GO:0006457) (Fig. 5H, all DEGs shown for spinal cord analysis).

Much like the cortical analysis, these biological processes revealed an evolving change over the course of the study from cellular responses from the initial trauma to a state of significant cellular misregulation. Analysis of this area of the spinal cord also illustrates the vulnerability of functionally connected distal regions to a focal trauma as was produced here. Furthermore, as in the cortical analysis, these misregulated biological processes are indicative of an ongoing pathological process showing signs of systemwide neurodegeneration.

## Discussion

The overarching hypothesis for this study was that injury-induced neurodegenerative sequalae from a focal cortical injury would impact functionally connected regions of the CNS distal to and separate from the primary injury site. We further hypothesized that these effects would persist and possibly increase over time. The prominent biomarker of TDP-43 mislocalization was selected as the primary biomarker to monitor the time course of neuronal neurodegeneration following a unilateral TBI of the motor cortex. RNAseq was also performed to determine transcriptional changes that occur at the subacute (i.e., 7, 14, and 28 DPI) and chronic (i.e., 120 and 180 DPI) timepoints following a TBI. Ultimately, both histochemical and transcriptomic data supported our initial hypothesis that TBI induces long-term neurodegenerative that emerge both acutely and chronically post-injury.

The histological analysis of the cortex rostral to and separate from the primary injury site revealed an overall significant increase incidence of neuronal TDP-43 mislocalization over time when compared to age-matched controls. This increase was observed in a cortical layer-dependent manner with significant amounts of TDP-43 mislocalization in cortical layers 2–3 and to a lesser extent layer 5, at subacute timepoints. In contrast, significant TDP-43 mislocalization in cortical layer 6 was not detected until 180 DPI.

This spatial and temporal pattern of TDP-43 mislocalization may be attributed to the susceptibility of the upper cortical layers 2–3 to local neuroinflammatory cues, or a change in cell-type specific cortical neuronal activity corresponding to the cortical depth or distance from the injury site [47]. The supragranular cortical layers are also primarily intracortical and may experience dysregulation due to hyperexcitation or the loss of inhibitory signaling [46, 47]. These events could include the sudden loss of connectivity or a sudden spike in neurotransmission, excitatory or inhibitory, thereby damaging the downstream neuron. In turn over time, deeper innervating cortical layers would be affected later in the pathological time-course following the TBI. This spatial pattern of TDP-43 mislocalization, following cortical connectivity structure, is comparable to degeneration in prion-like spreading in ALS [49]. Our data shows a similar neurodegenerative pattern that extends along the corticospinal tract to the functionally (synaptically) connected cervical spinal cord. Specifically, although a significant incidence of neuronal TDP-43 mislocalization was observed above naïve in the subacute phase, the 180 DPI chronic timepoint revealed a significant increase compared to all other timepoints.

NEUN was used as the mature neuronal marker for this study. Interestingly, we noted that the neurons affected by TDP-43 mislocalization typically also exhibited a cytosolic NEUN staining patterns. The neurons were not devoid of NEUN, yet the staining pattern was cytosolic in comparison to commonly observed NEUN nuclear localization. In 2009, Kim et. al., identified and defined NEUN neuronal marker as RBFOX3, an RNA-binding protein involved in the regulation of alternative splicing of pre-mRNA [50]. Kim further predicted 4 different isoforms but provided no information concerning the localization, nuclear or cytoplasmic, of the differing isoforms. Dredge and Jensen in 2011 used N-terminal flag-tags on differing isoforms of RBFOX3 constructs and found that one isoform labeled Fox3v1 was predominately nuclear, one isoform labeled Fox3v2 appeared exclusively nuclear, and one isoform labeled Fox3v3 appeared to be predominantly cytoplasmic. They further state that the only difference between Fox3v2 and Fox3v3 is the absence of a 14 amino acid C-terminal extension [51]. Lucas et. al., reported in 2013 that NEUN localization is altered in the context of a neurological disorders such as those observed in HIV related neurological disorders. They further posited that cytosolic isoform dominance was to downregulate NEUNs functions under pathological conditions [52]. While the cellular localization of NEUN was not quantified in this study, our observations further suggest that the detected TDP-43 nucleocytoplasmic mislocated cells exhibit further markers of neuronal pathology.

Our RNAseq analysis focused on identical areas assessed for TDP-43 proteinopathies in both the cortex, rostral to and separate from the primary injury site, as well as the functionally connected cervical spinal cord. For this analysis we initially chose only those transcripts that had a fold change of log2 > 0.585 (1.5 fold change) and a padj value < 0.05. This initial investigation was concerned with detecting transcriptional misregulation and the effects over time following a TBI. At the earliest timepoint (7 DPI), a modest level of significantly misregulated genes, both up and downregulated, was observed compared to naïve age-matched controls. This misregulation at 7 DPI may be attributed to acute injury response that then subsided over the subacute timepoints of 14 and 28 DPI timepoints. However, a major uptick in the number of misregulated genes was observed at the 120 and 180 DPI chronic timepoints. While the most pronounced effect was in the cortical tissue, the transcriptomic data for the spinal cord tissue followed the same basic patterns. Gene ontology analysis identified several significant biological processes that contribute to neurodegeneration [37, 38]. Both the cortical samples and the spinal cord samples are represented by the same major GO terms that include cellular signaling, response to stress or other stimulus, and regulation of gene expression and RNA processes that are most predominant at chronic timepoints. We acknowledge that the current RNAseq analysis was limited to bulk tissue analysis; future analysis with these data will include in-depth gene set enrichment analysis/pathway analysis and in silico deconvolution methods to infer or estimate cell type fractions from our samples (i.e., CIBERSORTx) [53].

Earlier studies probed the impact of TBI as a predisposing component in the development of neurodegenerative disease. Anderson et. al., looked at the effect of TBI on nuclear pore complex (NPC) and nucleocytoplasmic transport (NCT) proteins such as TDP-43 in *Drosophila*, rat, HEK293T cells and human post-mortem tissue [54]. In human post-mortem tissue of severe CTE, they reported elevated levels of Nucleoporin 62 (NUP62), a regulator of macromolecule flow between the nucleus and cytoplasm. In comparison, our DEG analysis found slight yet significant downregulation of NUP62 mRNA levels in subacute and chronic timepoints (subacute −0.194 log2 (−1.143 fold change, padj = 2.26e−02) and chronic −0.327 log2 (−1.254 fold change, padj = 1.77e−07). Gao et. al., showed in their study of mild and repeated TBI at 30 DPI in hippocampal tissue that aberrant production of TDP-43 following a TBI promotes tau phosphorylation and amyloid-β formation through the phosphorylation of Glycogen Synthase Kinase 3 Beta (GSK3β) and expression of Beta-Secretase 1 (BACE1) [48]. DEG in our study revealed no significant differences at any timepoints for GSK3β or BACE1 at subacute timepoints, but again found slight yet significant downregulation at chronic timepoints of 120 and 180 DPI (120 DPI at −0.111 log2 (−1.080 fold change), padj = 1.67e−02 and 180 DPI at −0.142 log2 (−1.103 fold change), padj = 7.32e−04). In contrast to these studies, Wiesner et. al., analyzed TDP-43 granules at up to 90 DPI in wild-type mice that had received a stab-injury to the motor cortex [55]. They reported that the nuclear pool of phospho-TDP-43 remained intact, at 3 DPI phospho-TDP-43 cytoplasmic granules appeared in ~ 6% of layer 5 neurons declining slightly by 7 DPI and returning to baseline amounts by 40 DPI. In our current study, TDP-43 mislocalization persisted and increased to the 180 DPI timepoint with prominent TDP-43 accumulations visible in the cytoplasm. We acknowledge the critical importance of assessing levels of phospho-TDP-43 in addition to TDP-43 mislocalization to fully assess TDP-43 proteinopathies; we note the absence of assessing phospho-TDP-43 as a limitation in this current study and plan to include the analysis in future studies. In comparisons to these studies and others (Jankovic et. al., Wang et. al.,), our data show an increasing neurodegenerative effect following a TBI and that these TBI associated neurodegenerative effects may be attributed to the creation of a chronic disease state [56, 32].

## Conclusion

As stated, this current IHC analysis used TDP-43 mislocalization as the primary indicator of neurodegenerative disease to determine the pervasive nature of neurodegenerative diseases following a TBI, additional analyses are needed to fully characterize the chronic neurodegenerative effects following a TBI using non-transgenic animal models. These studies would include: spatial proteomics analysis of the cortex as well as the spinal cord to provide further insights in the TDP-43 mislocalization patterns and additional data identifying the temporal and neuronal cell-types involved, behavioral and cognitive time-course analyses to identify parallels to the observed histological data, and finally, structural and phosphorylation analyses of TDP-43 cytoplasmic accumulations and solubility changes in TDP-43 cytoplasmic accumulations to compare them to pathological TDP-43 aggregates. We acknowledge additional limits of our study and the need for future studies. Specifically, additional analysis of brain regions that are not functionally connected to the primary injury site could provide insight into the synaptic progression we hypothesize versus possible global injury-induced changes. And lastly, analysis of additional neurodegenerative disease markers such as beta-amyloid, tau, or phosphor-tau is needed to further examine the broader basis of TBI related neurodegenerative effects. These analyses, which are beyond the design and scope of this study, will be instrumental in identifying novel therapeutic targets for mitigating TBI-induced neurodegeneration.

### Supplementary Information

**Additional file 1**. **Table S1.** Analysis of number of NEUN-positive neurons sampled between ipsilateral (injured) and contralateral (non-injured) cortical hemispheres in the unilateral TBI model at all analyzed time points. (SEM=standard error of the mean, *p* value=statistical significance, t=t-value, df=degrees of freedom, n=number of samples). **Table S2.** Percent of NEUN-positive neurons sampled that display indications of TDP-43 mislocalization over all time points for injured and naïve age-matched control groups. (SEM=standard error of the mean, n=number of samples). **Table S3.** Statistical analysis of injured versus naïve age-matched control group comparisons for NEUN-positive neuron counts at each timepoint analyzed. (SEM=standard error of the mean, *p* value=statistical significance, t=t-value, df=degrees of freedom, n=number of samples). **Table S4.** Statistical analysis of NEUN positive cells displaying TDP-43 nucleocytoplasmic mislocalization between the ipsilateral and contralateral cortices for all timepoints in injured and naïve controls. (SEM=standard error of the mean, *p* value=statistical significance, ns=not significant). **Table S5.** Statistical analysis of all timepoint comparisons between injured and naïve age-matched control groups for total cortical NEUN-positive neurons displaying nuclear TDP-43 mislocalization. (Adjusted *p* value=statistical significance, t=t-value, df=degrees of freedom, n=number of samples, ns=not significant) (Significance: *p<0.05, **p<0.01, ***p<0.001, ****p<0.0001). **Table S6.** Statistical analysis of all timepoint comparisons between injured and naïve age-matched control groups for cortical layer 2/3 NEUN-positive neurons displaying nuclear TDP-43 mislocalization. (Adjusted *p* value=statistical significance, t=t-value, df=degrees of freedom, n=number of samples, ns=not significant) (Significance: *p<0.05, **p<0.01, ***p<0.001, ****p<0.0001). **Table S7.** Statistical analysis of all timepoint comparisons between injured and naïve age-matched control groups for cortical layer 5 NEUN-positive neurons displaying nuclear TDP-43 mislocalization. (Adjusted *p* value=statistical significance, t=t-value, df=degrees of freedom, n=number of samples, ns=not significant) (Significance: *p<0.05, **p<0.01, ***p<0.001, ****p<0.0001). **Table S8.** Statistical analysis of all timepoint comparisons between injured and naïve age-matched control groups for cortical layer 6 NEUN-positive neurons displaying nuclear TDP-43 mislocalization. (Adjusted p value=statistical significance, t=t-value, df=degrees of freedom, n=number of samples) (Significance: *p<0.05, **p<0.01, ***p<0.001, ****p<0.0001). **Table S9.** Analysis of number of NEUN-positive neurons sampled between ipsilateral and contralateral spinal cord hemispheres in the unilateral TBI model at all analyzed time points. (SEM=standard error of the mean, *p* value=statistical significance, t=t-value, df=degrees of freedom, n=number of samples). **Table S10.** Statistical analysis of NEUN positive cells displaying TDP-43 nucleocytoplasmic mislocalization between the ipsilateral and contralateral spinal cord hemispheres for all timepoints in injured and naïve controls. (SEM=standard error of the mean, p value=statistical significance, t=t-value, df=degrees of freedom, n=number of samples, ns=not significant). **Table S11.** Statistical analysis of all timepoint comparisons between injured and naïve age-matched control groups for total spinal cord NEUN-positive neurons displaying nuclear TDP-43 mislocalization. (Adjusted *p* value=statistical significance, t=t-value, df=degrees of freedom, n=number of samples).

## Data Availability

The datasets used and/or analyzed during the current study available from the corresponding author on reasonable request.

## References

[1] Bekris LM, Yu C-E, Bird TD, Tsuang DW (2010). Genetics of Alzheimer Disease. J Geriatr Psychiatry Neurol.

[2] Amyotrophic lateral sclerosis: MedlinePlus Genetics. https://medlineplus.gov/genetics/condition/amyotrophic-lateral-sclerosis/. Accessed 28 Oct 2021

[3] Final-FTD-Genetics-Brochure-with-Cover-8–2–2012.pdf

[4] Chen H, Richard M, Sandler DP, Umbach DM, Kamel F (2007). Head injury and amyotrophic lateral sclerosis. Am J Epidemiol.

[5] Gupta R, Sen N (2016). Traumatic brain injury: a risk factor for neurodegenerative diseases. Rev Neurosci.

[6] LoBue C, Cullum CM, Didehbani N, Yeatman K, Jones B, Kraut MA, Hart J (2018). Neurodegenerative dementias after traumatic brain injury. JNP.

[7] Dewan MC, Rattani A, Gupta S, Baticulon RE, Hung Y-C, Punchak M, Agrawal A, Adeleye AO, Shrime MG, Rubiano AM, Rosenfeld JV, Park KB (2019). Estimating the global incidence of traumatic brain injury. J Neurosurg.

[8] MacFarlane MP, Glenn TC (2015). Neurochemical cascade of concussion. Brain Inj.

[9] Prins M, Greco T, Alexander D, Giza CC (2013). The pathophysiology of traumatic brain injury at a glance. Dis Model Mech.

[10] Combs HL, Jones TA, Kozlowski DA, Adkins DL (2016). Combinatorial motor training results in functional reorganization of remaining motor cortex after controlled cortical impact in rats. J Neurotrauma.

[11] Davies M, Jacobs A, Brody DL, Friess SH (2018). Delayed hypoxemia after traumatic brain injury exacerbates long-term behavioral deficits. J Neurotrauma.

[12] Golub VM, Reddy DS (2022). Contusion brain damage in mice for modelling of post-traumatic epilepsy with contralateral hippocampus sclerosis: comprehensive and longitudinal characterization of spontaneous seizures, neuropathology, and neuropsychiatric comorbidities. Exp Neurol.

[13] Hånell A, Clausen F, Djupsjö A, Vallstedt A, Patra K, Israelsson C, Larhammar M, Björk M, Paixão S, Kullander K, Marklund N (2012). Functional and histological outcome after focal traumatic brain injury is not improved in conditional EphA4 knockout mice. J Neurotrauma.

[14] Scotter EL, Chen H-J, Shaw CE (2015). TDP-43 proteinopathy and ALS: insights into disease mechanisms and therapeutic targets. Neurotherapeutics.

[15] Stelfa G, Svalbe B, Vavers E, Duritis I, Dambrova M, Zvejniece L (2022). Moderate traumatic brain injury triggers long-term risks for the development of peripheral pain sensitivity and depressive-like behavior in mice. Front Neurol.

[16] Huang W, Zhou Y, Tu L, Ba Z, Huang J, Huang N, Luo Y (2020). TDP-43: from Alzheimer’s disease to limbic-predominant age-related TDP-43 encephalopathy. Front Mol Neurosci.

[17] Prasad A, Bharathi V, Sivalingam V, Girdhar A, Patel BK (2019). Molecular mechanisms of TDP-43 misfolding and pathology in amyotrophic lateral sclerosis. Front Mol Neurosci.

[18] Jo M, Lee S, Jeon Y-M, Kim S, Kwon Y, Kim H-J (2020). The role of TDP-43 propagation in neurodegenerative diseases: integrating insights from clinical and experimental studies. Exp Mol Med.

[19] Ling S-C, Polymenidou M, Cleveland DW (2013). Converging mechanisms in ALS and FTD: disrupted RNA and protein homeostasis. Neuron.

[20] Suk TR, Rousseaux MWC (2020). The role of TDP-43 mislocalization in amyotrophic lateral sclerosis. Mol Neurodegeneration.

[21] Blennow K, Hardy J, Zetterberg H (2012). The neuropathology and neurobiology of traumatic brain injury. Neuron.

[22] Heyburn L, Sajja VSSS, Long JB (2019). The role of TDP-43 in military-relevant TBI and chronic neurodegeneration. Front Neurol.

[23] McKee AC, Gavett BE, Stern RA, Nowinski CJ, Cantu RC, Kowall NW, Perl DP, Hedley-Whyte ET, Price B, Sullivan C, Morin P, Lee H-S, Kubilus CA, Daneshvar DH, Wulff M, Budson AE (2010). TDP-43 proteinopathy and motor neuron disease in chronic traumatic encephalopathy. J Neuropathol Exp Neurol.

[24] McKee AC, Stein TD, Nowinski CJ, Stern RA, Daneshvar DH, Alvarez VE, Lee H-S, Hall G, Wojtowicz SM, Baugh CM, Riley DO, Kubilus CA, Cormier KA, Jacobs MA, Martin BR, Abraham CR, Ikezu T, Reichard RR, Wolozin BL, Budson AE, Goldstein LE, Kowall NW, Cantu RC (2013). The spectrum of disease in chronic traumatic encephalopathy. Brain.

[25] The Neuropathology of Chronic Traumatic Encephalopathy. 10.1111/bpa.12248 10.1111/bpa.12248 PMC4526170 25904048

[26] McKee AC, Gavett BE, Stern RA, Nowinski CJ, Cantu RC, Kowall NW, Perl DP, Hedley-Whyte ET, Price B, Sullivan C, Morin P, Lee H-S, Kubilus CA, Daneshvar DH, Wulff M, Budson AE (2010). TDP-43 proteinopathy and motor neuron disease in chronic traumatic encephalopathy. J Neuropathol Exp Neurol.

[27] Huang C-H, Lin C-W, Lee Y-C, Huang C-Y, Huang R-Y, Tai Y-C, Wang K-W, Yang S-N, Sun Y-T, Wang H (2018). Is traumatic brain injury a risk factor for neurodegeneration? A meta-analysis of population-based studies. BMC Neurol.

[28] Janković T, Dolenec P, Bumber JR, Gržeta N, Kriz J, Župan G, Pilipović K (2021). Differential expression patterns of TDP-43 in single moderate versus repetitive mild traumatic brain injury in mice. Int J Mol Sci.

[29] Kahriman A, Bouley J, Smith TW, Bosco DA, Woerman AL, Henninger N (2021). Mouse closed head traumatic brain injury replicates the histological tau pathology pattern of human disease: characterization of a novel model and systematic review of the literature. Acta Neuropathol Commun.

[30] Rajič Bumber J, Pilipović K, Janković T, Dolenec P, Gržeta N, Križ J, Župan G (2021). Repetitive traumatic brain injury is associated with TDP-43 alterations, neurodegeneration, and glial activation in mice. J Neuropathol Exp Neurol.

[31] Tan XL, Sun M, Brady RD, Liu S, Llanos R, Cheung S, Wright DK, Casillas-Espinosa PM, Sashindranath M, O’Brien TJ, McDonald SJ, Turner BJ, Shultz SR (2019). Transactive response DNA-binding protein 43 abnormalities after traumatic brain injury. J Neurotrauma.

[32] Wang H-K, Lee Y-C, Huang C-Y, Liliang P-C, Lu K, Chen H-J, Li Y-C, Tsai K-J (2015). Traumatic brain injury causes frontotemporal dementia and TDP-43 proteolysis. Neuroscience.

[33] Zhang J, Teng Z, Song Y, Hu M, Chen C (2015). Inhibition of monoacylglycerol lipase prevents chronic traumatic encephalopathy-like neuropathology in a mouse model of repetitive mild closed head injury. J Cereb Blood Flow Metab.

[34] Cole JT, Yarnell A, Kean WS, Gold E, Lewis B, Ren M, McMullen DC, Jacobowitz DM, Pollard HB, O’Neill JT, Grunberg NE, Dalgard CL, Frank JA, Watson WD (2011). Craniotomy: true sham for traumatic brain injury, or a sham of a sham?. J Neurotrauma.

[35] Allen Reference Atlas - Mouse Brain [brain atlas]. Available from atlas.brain-map.org

[36] MouseLight Neuron Browser. http://ml-neuronbrowser.janelia.org/. Accessed 27 Jun 2023

[37] Ramanan VK, Saykin AJ (2013). Pathways to neurodegeneration: mechanistic insights from GWAS in Alzheimer’s disease, Parkinson’s disease, and related disorders. Am J Neurodegener Dis.

[38] Ruffini N, Klingenberg S, Schweiger S, Gerber S (2020). Common factors in neurodegeneration: a meta-study revealing shared patterns on a multi-omics scale. Cells.

[39] Bharadwaj VN, Copeland C, Mathew E, Newbern J, Anderson TR, Lifshitz J, Kodibagkar VD, Stabenfeldt SE (2020). Sex-dependent macromolecule and nanoparticle delivery in experimental brain injury. Tissue Eng Part A.

[40] Martinez BI, Mousa GA, Fleck K, MacCulloch T, Diehnelt CW, Stephanopoulos N, Stabenfeldt SE Uncovering temporospatial sensitive TBI targeting strategies via in vivo phage display. Sci Adv 8:eabo5047. 10.1126/sciadv.abo5047 10.1126/sciadv.abo5047 PMC9307250 35867794

[41] Siebold L, Obenaus A, Goyal R (2018). Criteria to define mild, moderate, and severe traumatic brain injury in the mouse controlled cortical impact model. Exp Neurol.

[42] Franklin KBJ, Paxinos G (2008). The mouse brain in stereotaxic coordinates.

[43] Schneider CA, Rasband WS, Eliceiri KW (2012). NIH Image to ImageJ: 25 years of image analysis. Nat Methods.

[44] Mi H, Ebert D, Muruganujan A, Mills C, Albou L-P, Mushayamaha T, Thomas PD (2021). PANTHER version 16: a revised family classification, tree-based classification tool, enhancer regions and extensive API. Nucl Acids Res.

[45] Ayala YM, Zago P, D’Ambrogio A, Xu Y-F, Petrucelli L, Buratti E, Baralle FE (2008). Structural determinants of the cellular localization and shuttling of TDP-43. J Cell Sci.

[46] Carron SF, Alwis DS, Rajan R (2016). Traumatic brain injury and neuronal functionality changes in sensory cortex. Front Syst Neurosci.

[47] Carron SF, Yan EB, Alwis DS, Rajan R (2016). Differential susceptibility of cortical and subcortical inhibitory neurons and astrocytes in the long term following diffuse traumatic brain injury. J Compar Neurol.

[48] Gao F, Hu M, Zhang J, Hashem J, Chen C (2022). TDP-43 drives synaptic and cognitive deterioration following traumatic brain injury. Acta Neuropathol.

[49] Polymenidou M, Cleveland DW (2011). The Seeds of Neurodegeneration: Prion-like Spreading in ALS. Cell.

[50] Kim KK, Adelstein RS, Kawamoto S (2009). Identification of neuronal nuclei (NeuN) as Fox-3, a new member of the Fox-1 gene family of splicing factors. J Biol Chem.

[51] Dredge BK, Jensen KB (2011) NeuN/Rbfox3 nuclear and cytoplasmic isoforms differentially regulate alternative splicing and nonsense-mediated decay of Rbfox2. PLOS ONE 6:e21585. 10.1371/journal.pone.0021585 10.1371/journal.pone.0021585 PMC3126832 21747913

[52] Lucas C-H, Calvez M, Babu R, Brown A (2014). Altered subcellular localization of the NeuN/Rbfox3 RNA splicing factor in HIV-associated neurocognitive disorders (HAND). Neurosci Lett.

[53] Newman AM, Steen CB, Liu CL, Gentles AJ, Chaudhuri AA, Scherer F, Khodadoust MS, Esfahani MS, Luca BA, Steiner D, Diehn M, Alizadeh AA (2019). Determining cell type abundance and expression from bulk tissues with digital cytometry. Nat Biotechnol.

[54] Anderson EN, Morera AA, Kour S, Cherry JD, Ramesh N, Gleixner A, Schwartz JC, Ebmeier C, Old W, Donnelly CJ, Cheng JP, Kline AE, Kofler J, Stein TD, Pandey UB (2021). Traumatic injury compromises nucleocytoplasmic transport and leads to TDP-43 pathology. Elife.

[55] Wiesner D, Tar L, Linkus B, Chandrasekar A, olde Heuvel F, Dupuis L, Tsao W, Wong PC, Ludolph A, Roselli F (2018). Reversible induction of TDP-43 granules in cortical neurons after traumatic injury. Exp Neurol.

[56] Janković T, Dolenec P, Rajič Bumber J, Gržeta N, Kriz J, Župan G, Pilipović K (2021). Differential expression patterns of TDP-43 in single moderate versus repetitive mild traumatic brain injury in mice. Int J Mol Sci.

