# Supplementary material for: Traumatic brain injury induces TDP-43 mislocalization and neurodegenerative effects in tissue distal to the primary injury site in a non-transgenic mouse
Source: Acta Neuropathol Commun. 2023 Aug 22;11:137. doi: 10.1186/s40478-023-01625-7 (PMC10463884; doi:10.1186/s40478-023-01625-7)
Supplement: Supplementary file 1 — Additional file 1. Table S1. Analysis of number of NEUN-positive neurons sampled between ipsilateral (injured) and contralateral (non-injured) cortical hemispheres in the unilateral TBI model at all analyzed time points. (SEM=standard error of the mean, p value=statistical significance, t=t-value, df=degrees of freedom, n=number of samples). Table S2. Percent of NEUN-positive neurons sampled that display indications of TDP-43 mislocalization over all time points for injured and naïve age-matched control groups. (SEM=standard error of the mean, n=number of samples). Table S3. Statistical analysis of injured versus naïve age-matched control group comparisons for NEUN-positive neuron counts at each timepoint analyzed. (SEM=standard error of the mean, p value=statistical significance, t=t-value, df=degrees of freedom, n=number of samples). Table S4. Statistical analysis of NEUN positive cells displaying TDP-43 nucleocytoplasmic mislocalization between the ipsilateral and contralateral cortices for all timepoints in injured and naïve controls. (SEM=standard error of the mean, p value=statistical significance, ns=not significant). Table S5. Statistical analysis of all timepoint comparisons between injured and naïve age-matched control groups for total cortical NEUN-positive neurons displaying nuclear TDP-43 mislocalization. (Adjusted p value=statistical significance, t=t-value, df=degrees of freedom, n=number of samples, ns=not significant) (Significance: *p<0.05, **p<0.01, ***p<0.001, ****p<0.0001). Table S6. Statistical analysis of all timepoint comparisons between injured and naïve age-matched control groups for cortical layer 2/3 NEUN-positive neurons displaying nuclear TDP-43 mislocalization. (Adjusted p value=statistical significance, t=t-value, df=degrees of freedom, n=number of samples, ns=not significant) (Significance: *p<0.05, **p<0.01, ***p<0.001, ****p<0.0001). Table S7. Statistical analysis of all timepoint comparisons between injured and naïve age-matched c [file 40478_2023_1625_MOESM1_ESM.pdf]

Supplemental Table 1

	Ipsilateral		Contralateral					
	**Avg**	**± SEM**	**Avg**	**± SEM**	***p* value**	**t**	**df**	**n**
**7 DPI**	797.0	101.90	760.5	51.51	p=.7418	0.35	5.00	4/3
**14 DPI**	749.3	48.39	734.0	70.26	p=.8640	0.18	6.00	4/4
**28 DPI**	875.8	93.07	767.5	32.42	p=.3142	1.10	6.00	4/4
**120 DPI**	713.0	48.40	660.0	57.38	p=.5067	0.71	6.00	4/4
**180 DPI**	725.3	54.65	725.3	85.42	p=>.9999	0.00	6.00	4/4
**7-28 Control**	879.5	145.50	880.0	130.94	p=.9982	0.00	3.00	2/3
**120 Control**	677.5	62.01	736.3	92.09	p=.6157	0.53	6.00	4/4
**180 Control**	697.8	50.31	676.5	80.59	p=.8304	0.22	6.00	4/4

Analysis of number of NEUN-positive neurons sampled between ipsilateral (injured) and contralateral (non-injured) cortical hemispheres in the unilateral TBI model at all analyzed time points. (SEM=standard error of the mean, p value=statistical significance, t=t-value, df=degrees of freedom, n=number of samples)

Supplemental Table 2

	Average	SEM	n
**7 DPI:Injured**	8.55	1.33	3
**14 DPI:Injured**	6.12	1.04	4
**28 DPI:Injured**	7.48	1.23	4
**120 DPI:Injured**	6.46	0.95	4
**180 DPI:Injured**	13.27	1.32	4
**7-28 DPI: Control**	2.44	0.69	2
**120 DPI: Control**	3.37	0.61	4
**180DPI: Control**	6.22	2.21	4

Percent of NEUN-positive neurons sampled that display indications of TDP-43 mislocalization over all time points for injured and naïve age-matched control groups. (SEM=standard error of the mean, n=number of samples)

Supplemental Table 3

	Injured		Control					
	**Avg**	**± SEM**	**Avg**	**± SEM**	***p* value**	**t**	**df**	**n**
**7 DPI:Injured vs. 7 DPI:Control**	792.7	71.53	917.0	166.00	>0.9999	1.23	27.00	3/2
**14 DPI:Injured vs. 14 DPI:Control**	741.6	58.01	917.0	166.00	>0.9999	1.23	27.00	4/2
**28 DPI:Injured vs. 28 DPI:Control**	821.6	46.91	917.0	166.00	>0.9999	1.23	27.00	4/2
**120 DPI:Injured vs. 120 DPI:Control**	686.5	51.30	706.9	74.77	>0.9999	1.23	27.00	4/4
**180 DPI:Injured vs. 180 DPI:Control**	725.3	68.28	687.1	62.06	>0.9999	1.23	27.00	4/4

Statistical analysis of injured vs. naïve age-matched control group comparisons for NEUN-positive neuron counts at each timepoint analyzed. (SEM=standard error of the mean, p value=statistical significance, t=t-value, df=degrees of freedom, n=number of samples)

Supplemental Table 4

	Ipsilateral		Contralateral			
	**Avg**	**± SEM**	**Avg**	**± SEM**	***p* value**	**Summary**
**7 DPI**	8.44	0.61	7.32	2.70	p=.5824	ns
**14 DPI**	5.33	1.20	6.17	1.56	p=.6412	ns
**28 DPI**	7.67	2.59	6.19	1.98	p=.4625	ns
**120 DPI**	6.35	2.12	5.63	0.81	p=.6050	ns
**180 DPI**	11.72	3.03	11.59	1.62	p=.9478	ns
**7-28 Control**	1.90	0.75	2.41	0.74	p=.6020	ns
**120 Control**	3.52	1.91	2.92	1.01	p=.6506	ns
**180 Control**	6.08	3.08	5.41	3.50	p=.8102	ns

Statistical analysis of NEUN positive cells displaying TDP-43 nucleocytoplasmic mislocalization between the ipsilateral and contralateral cortices for all timepoints in injured and naïve controls. (SEM=standard error of the mean, p value=statistical significance, ns=not significant)

Supplemental Table 5

	Summary	Adjusted *p* Value	t	df	n
**7 DPI:Injured vs. 7 DPI:Control**	***	0.0002	5.763	27	3/2
**7 DPI:Injured vs. 180 DPI:Injured**	ns	0.2687	2.984	27	3/4
**14 DPI:Injured vs. 14 DPI:Control**	***	0.0002	5.763	27	4/2
**14 DPI:Injured vs. 180 DPI:Injured**	**	0.0088	4.308	27	4/4
**28 DPI:Injured vs. 28 DPI:Control**	***	0.0002	5.763	27	4/2
**28 DPI:Injured vs. 180 DPI:Injured**	ns	0.0535	3.623	27	4/4
**120 DPI:Injured vs. 120 DPI:Control**	***	0.0002	5.763	27	4/4
**120 DPI:Injured vs. 180 DPI:Injured**	*	0.0210	3.981	27	4/4
**180 DPI:Injured vs. 180 DPI:Control**	***	0.0002	5.763	27	4/4

Table also includes comparisons between each timepoint analyzed and the 180 DPI timepoint

Statistical analysis of all timepoint comparisons between injured and naïve age-matched control groups for total cortical NEUN-positive neurons displaying nuclear TDP-43 mislocalization. (Adjusted p value=statistical significance, t=t-value, df=degrees of freedom, n=number of samples, ns=not significant) (Significance: *p<0.05, **p<0.01, ***p<0.001, ****p<0.0001)

Supplemental Table 6

	Summary	Adjusted P Value	t	df	n
**7 DPI:Injured vs. 7 DPI:Control**	***	0.0002	5.821	27	3/2
**7 DPI:Injured vs. 180 DPI:Injured**	ns	0.9781	2.436	27	3/4
**14 DPI:Injured vs. 14 DPI:Control**	***	0.0002	5.821	27	4/2
**14 DPI:Injured vs. 180 DPI:Injured**	**	0.0055	4.479	27	4/4
**28 DPI:Injured vs. 28 DPI:Control**	***	0.0002	5.821	27	4/2
**28 DPI:Injured vs. 180 DPI:Injured**	ns	0.0811	3.462	27	4/4
**120 DPI:Injured vs. 120 DPI:Control**	***	0.0002	5.821	27	4/4
**120 DPI:Injured vs. 180 DPI:Injured**	ns	0.1048	3.361	27	4/4
**180 DPI:Injured vs. 180 DPI:Control**	***	0.0002	5.821	27	4/4

Table also includes comparisons between each timepoint analyzed and the 180 DPI timepoint

Statistical analysis of all timepoint comparisons between injured and naïve age-matched control groups for cortical layer 2/3 NEUN-positive neurons displaying nuclear TDP-43 mislocalization. (Adjusted p value=statistical significance, t=t-value, df=degrees of freedom, n=number of samples, ns=not significant) (Significance: *p<0.05, **p<0.01, ***p<0.001, ****p<0.0001)

Supplemental Table 7

	Summary	Adjusted P Value	t	df	n
**7 DPI:Injured vs. 7 DPI:Control**	****	<0.0001	6.539	31	4/3
**7 DPI:Injured vs. 180 DPI:Injured**	ns	0.7402	2.536	31	4/4
**14 DPI:Injured vs. 14 DPI:Control**	****	<0.0001	6.539	31	4/3
**14 DPI:Injured vs. 180 DPI:Injured**	ns	0.0520	3.580	31	4/4
**28 DPI:Injured vs. 28 DPI:Control**	****	<0.0001	6.653	31	4/3
**28 DPI:Injured vs. 180 DPI:Injured**	ns	0.4081	2.784	31	4/4
**120 DPI:Injured vs. 120 DPI:Control**	****	<0.0001	6.539	31	4/4
**120 DPI:Injured vs. 180 DPI:Injured**	*	0.0393	3.683	31	4/4
**180 DPI:Injured vs. 180 DPI:Control**	****	<0.0001	6.539	31	4/4

Table also includes comparisons between each timepoint analyzed and the 180 DPI timepoint

Statistical analysis of all timepoint comparisons between injured and naïve age-matched control groups for cortical layer 5 NEUN-positive neurons displaying nuclear TDP-43 mislocalization. (Adjusted p value=statistical significance, t=t-value, df=degrees of freedom, n=number of samples, ns=not significant) (Significance: *p<0.05, **p<0.01, ***p<0.001, ****p<0.0001)

Supplemental Table 8

	Summary	Adjusted P Value	t	df	n
**7 DPI:Injured vs. 7 DPI:Control**	*	0.0234	3.872	31	4/3
**7 DPI:Injured vs. 180 DPI:Injured**	**	0.0029	4.610	31	4/4
**14 DPI:Injured vs. 14 DPI:Control**	*	0.0234	3.872	31	4/3
**14 DPI:Injured vs. 180 DPI:Injured**	**	0.0076	4.274	31	4/4
**28 DPI:Injured vs. 28 DPI:Control**	*	0.0234	3.872	31	4/3
**28 DPI:Injured vs. 180 DPI:Injured**	**	0.0031	4.587	31	4/4
**120 DPI:Injured vs. 120 DPI:Control**	*	0.0234	3.872	31	4/4
**120 DPI:Injured vs. 180 DPI:Injured**	**	0.0075	4.281	31	4/4
**180 DPI:Injured vs. 180 DPI:Control**	*	0.0234	3.872	31	4/4

Table also includes comparisons between each timepoint analyzed and the 180 DPI timepoint

Statistical analysis of all timepoint comparisons between injured and naïve age-matched control groups for cortical layer 6 NEUN-positive neurons displaying nuclear TDP-43 mislocalization. (Adjusted p value=statistical significance, t=t-value, df=degrees of freedom, n=number of samples) (Significance: *$p<0.05$, **$p<0.01$, ***$p<0.001$, ****$p<0.0001$)

Supplemental Table 9

	Ipsilateral		Contralateral					
	**Avg**	**± SEM**	**Avg**	**± SEM**	**p value**	**t**	**df**	**n**
**7 DPI:Injured**	109.3	5.31	106.5	4.44	0.7051	0.40	6.00	4/4
**14 DPI:Injured**	102.3	5.57	107.5	7.32	0.5890	0.57	6.00	4/4
**28 DPI:Injured**	125.0	5.34	130.3	4.33	0.4739	0.76	6.00	4/4
**120 DPI:Injured**	115.5	1.19	125.3	2.66	0.1550	3.35	6.00	4/3
**180 DPI:Injured**	109.8	9.35	105.3	4.61	0.6810	0.43	6.00	4/4
**7-28 DPI: Control**	122.3	6.80	121.3	7.51	0.9246	0.10	6.00	4/4
**120 DPI: Control**	157.3	7.27	159.3	11.84	0.8925	0.14	4.00	4/4
**180DPI: Control**	115.8	5.25	117.8	8.20	0.8440	0.21	6.00	4/4

Naïve age-matched control groups were treated and sampled in the same manner as injured groups to maintain continuity

Analysis of number of NEUN-positive neurons sampled between ipsilateral and contralateral spinal cord hemispheres in the unilateral TBI model at all analyzed time points. Naïve age-matched control groups were treated and sampled in the same manner as injured groups to maintain continuity. (SEM=standard error of the mean, p value=statistical significance, t=t-value, df=degrees of freedom, n=number of samples)

Table 10

	Ipsilateral		Contralateral			
	**Avg**	**± SEM**	**Avg**	**± SEM**	**p value**	**Summary**
**7 DPI**	12.20	1.68	10.55	0.87	p=.3293	ns
**14 DPI**	9.09	2.89	8.07	1.58	p=.7661	ns
**28 DPI**	9.33	2.34	10.90	3.12	p=.7002	ns
**120 DPI**	5.48	1.58	6.50	0.75	p=.5814	ns
**180 DPI**	16.56	3.09	16.96	4.52	p=.9437	ns
**7-28 Control**	3.48	1.00	2.65	0.43	p=.4749	ns
**120 Control**	2.68	1.20	1.92	0.09	p=.5606	ns
**180 Control**	7.30	0.73	6.98	1.61	p=.8593	ns

Statistical analysis of NEUN positive cells displaying TDP-43 nucleocytoplasmic mislocalization between the ipsilateral and contralateral spinal cord hemispheres for all timepoints in injured and naïve controls. (SEM=standard error of the mean, p value=statistical significance, t=t-value, df=degrees of freedom, n=number of samples, ns=not significant)

Table 11

	Adjusted P Value	t	df	n
**7 DPI:Injured vs. 7 DPI:Control**	<0.0001	6.151	33	4/4
**7 DPI:Injured vs. 180 DPI:Injured**	0.4946	2.695	33	4/4
**14 DPI:Injured vs. 14 DPI:Control**	<0.0001	6.151	33	4/4
**14 DPI:Injured vs. 180 DPI:Injured**	0.0625	3.491	33	4/4
**28 DPI:Injured vs. 28 DPI:Control**	<0.0001	6.151	33	4/4
**28 DPI:Injured vs. 180 DPI:Injured**	0.2001	3.054	33	4/4
**120 DPI:Injured vs. 120 DPI:Control**	<0.0001	6.151	33	4/3
**120 DPI:Injured vs. 180 DPI:Injured**	0.0045	4.419	33	4/4
**180 DPI:Injured vs. 180 DPI:Control**	<0.0001	6.151	33	4/4

Table also includes comparisons between each timepoint analyzed and the 180 DPI timepoint

Statistical analysis of all timepoint comparisons between injured and naïve age-matched control groups for total spinal cord NEUN-positive neurons displaying nuclear TDP-43 mislocalization. (Adjusted p value=statistical significance, t=t-value, df=degrees of freedom, n=number of samples)
